# Ger-e-Tec home monitoring: a new home telemonitoring project for geriatric disorders

**DOI:** 10.25122/jml-2023-0062

**Published:** 2023-08

**Authors:** Abrar-Ahmad Zulfiqar, Mohamed Hajjam, Jawad Hajjam, Sylvie Erve

**Affiliations:** 1 Internal Medicine Unit, University Hospital of Strasbourg, Strasbourg, France; 2 Predimed Technology Society, Schiltigheim, France; 3 CENTICH, Angers, France

## DEAR EDITOR,

We present GER-e-TEC™ (Geriatrics and e-Technology) home monitoring study, a project offering a new type of care for older people in their residences. This initiative aims to anticipate acute episodes in patients and delay the progression of health deterioration, thereby enhancing their quality of life. The GER-e-TEC™ project aims to provide these patients with diverse needs, as well as medical (treating physicians) and paramedical (liberal nurses, etc.) teams with telemedicine tools, allowing protocolized and personalized, non-intrusive monitoring [1-3].

This will be a prospective, observational, non-randomized study taking place in the living environment of patients in the Philémon series [4]. The PHILEMON project is an innovative system for home living for increasingly dependent elderly persons (experimentation with innovative systems for home living in the French departments of Loire-Atlantique, Maine et Loire, Mayenne, Sarthe and Vendée). To improve geriatric monitoring at home, PHILEMON will be enhanced by an experiment based on an initial assessment performed with the ICOPE tool (Integrated Care for Older People) [5] supplemented by the GER-e-TEC tool, adapted to the patient's health status for early detection and to provide alerts to enable health professionals to optimize patient care. This experiment will be offered to the entire PHILEMON active file with a goal of including 30 elderly participants over a period of three years. This initiative will provide personalized monitoring of geriatric risks to improve the quality of life of elderly individuals in situations of functional decline due to dependence or chronic illness and to avoid unnecessary hospitalizations. This monitoring is adapted to the patient's health status for early detection and to provide alerts to enable health professionals to optimize the management of patients using the GER-e-TEC platform, which has been widely tested in hospitals and at home and featured in several publications. Inclusion criteria are elderly and dependent patients (Iso Resource Group 1-4) over 65 years, belonging to the Philemon series.

The paramedical teams involved in the daily monitoring of the elderly patient will ensure the weekly collection of hemodynamic measurements, such as blood pressure, heart rate, oxygen saturation, and capillary glycemia for patients with diabetes, facilitated by connected sensors. Physical activity will be measured through wearable pedometers.

The platform will incorporate questionnaires aimed at addressing primary geriatric risks on a weekly or monthly basis. This comprehensive approach covers factors such as constipation (the frequency of daily stools), dehydration (the appreciation of the oral mucosa), iatrogenic risk, heart failure (the EPOF questionnaire for patients known to have chronic heart disease), quality of sleep (neuro- and psycho-behavioral disorders) and the level of bed rest (bedsores/physical activity) contributing to the holistic understanding of each patient's condition. We have not found comparable studies in the scientific literature focusing on implementing home telemonitoring specifically addressing the primary geriatric risks and associated comorbidities such as heart failure or diabetes.

Nurses and caregivers, as well as family caregivers, have the free tools and sensors provided by the Predimed society. The data recorded by the sensors or filled in using the integrated questionnaires is then analyzed by the coordination unit if alerts are issued. Our telesurveillance project relies on various medical and paramedical teams acting in synergy. The coordination unit, made up of a Philémon nurse and a geriatrician, analyzes the data regularly, processes the alerts reported by the system by systematically checking their relevance, and constitutes a real relay between the elderly patient remotely monitored, the paramedical teams involved in their daily life and the attending physician. The coordination cell will make it possible to follow the patient page, adapt the treatments if necessary, and be a source of proposals in collaboration with the general practitioner with the implementation of accompanying measures such as the recommendation of an increase in physical activity, prevention of bedsores, and dietary monitoring.

**Figure 1:**
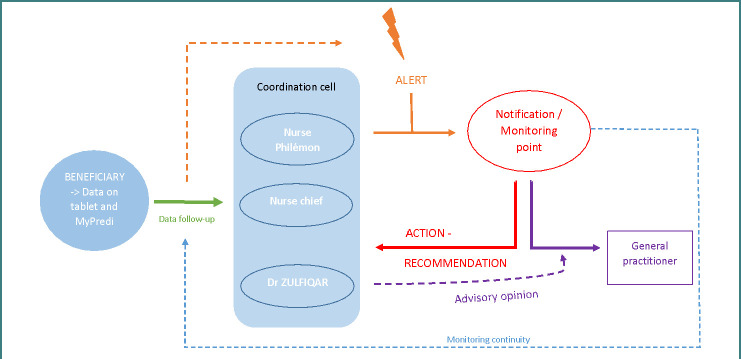
The Ger-e-Tec home monitoring project

Figure 1 explains the entire process and functioning of the Ger-e-Tec home monitoring project. This project started in June 2022, and we have included 11 patients since then. The objective is to include 30 patients before July 2024. The results of these series will be analyzed and communicated later.
